# The relations between different components of intolerance of uncertainty and symptoms of generalized anxiety disorder: a network analysis

**DOI:** 10.1186/s12888-021-03455-0

**Published:** 2021-09-10

**Authors:** Lei Ren, Zihan Wei, Ye Li, Long-Biao Cui, Yifei Wang, Lin Wu, Xinyi Wei, Jiaxi Peng, Kuiliang Li, Yinchuan Jin, Fengzhan Li, Qun Yang, Xufeng Liu

**Affiliations:** 1 Department of Military Medical Psychology, Air Force Medical University, Xi’an, 710032 China; 2 Department of Neurology, Xijing Hospital, Air Force Medical University, Xi’an, 710032 China; 3 Psychological counseling center, Xijing University, Xi’an, 710100 China; 4 Department of Psychology, Renmin University of China, Beijing, 100000 China; 5 College of Teachers, Chengdu University, Chengdu, 610106 China; 6 Department of Psychology, Army Medical University, Chongqing, 400038 China

**Keywords:** Generalized anxiety disorder, Intolerance of uncertainty, Worry, Network analysis

## Abstract

**Background:**

Intolerance of uncertainty (IU) is considered as a specific risk factor in the development and maintenance of generalized anxiety disorder (GAD). Yet, researches have investigated the relations between IU and GAD (or worry) using total scores on self-report measures. This ignores that there are different components exist in IU and the heterogeneity of GAD symptoms. In the present study, we explored the relations among different components of IU and symptoms of GAD.

**Methods:**

A dimensional approach which take individual differences into consideration in different components of IU along a full range of normal to abnormal symptom severity levels of GAD were used in this study. Components of IU were measured by 12-item Intolerance of Uncertainty Scale and symptoms of GAD were measured by Generalized Anxiety Disorder 7-Item Questionnaire. Regularized partial-correlation network was estimated using cross-sectional data from 624 university students.

**Results:**

Four strongest edges are between components of IU, like “Unforeseen events upset me greatly” and “It frustrates me not having all the information I need”. Two strongest edges are between symptoms of GAD, like “Being so restless that it is hard to sit still” and “Feeling afraid as if something awful might happen”. Symptom “Worrying too much about different things” and component “It frustrates me not having all the information I need” have the highest expected influences in the present network. In the community of IU, component “It frustrates me not having all the information I need” has the highest bridge expected influence. And in the community of GAD, symptoms “Worrying too much about different things” and “Not being able to stop or control worrying” have the highest bridge expected influence.

**Conclusions:**

This study reveals potential pathways between different components of IU and various symptoms of GAD. Understanding how putative risk factors such as different components of IU are related to symptoms of GAD may provide some references for related preventions and interventions, such as targeting component “It frustrates me not having all the information I need” may be more effective at reducing symptoms of GAD than targeting other components of IU.

**Supplementary Information:**

The online version contains supplementary material available at 10.1186/s12888-021-03455-0.

## Introduction

With the lifetime prevalence ranging from 4.3 to 5.9%, generalized anxiety disorder (GAD) is one of the most common mental health problems all over the world [1]. Characterized by excessive and uncontrollable worry about a series of events or activities lasting for at least 6 months, GAD often accompanies with other nonspecific psychological and physical symptoms [2]. Individuals with GAD have considerable role impairment and a high comorbidity with depression [3]. If GAD is not treated promptly, the prognosis is poor [4]. Therefore, it is important to identify the developing and maintaining factors for GAD to improve existing intervention strategies.

Intolerance of uncertainty (IU), the “individual’s dispositional incapacity to endure the aversive response triggered by the perceived absence of salient, key, or sufficient information, and sustained by the associated perception of uncertainty” [5], is considered to be a specific risk factor or cognitive vulnerability in the development and maintenance of anxiety disorders [6, 7]. To better explain the relationship between IU and the psychopathology of anxiety, the most comprehensive conceptual model was developed, which was designed primarily to explain the symptoms of GAD [8]. A recent meta-analysis article found that the strengths of association between IU and GAD symptoms is significantly higher than all other syndromes (i.e., depression, social anxiety disorder, panic/agoraphobia, obsessive compulsive disorder, eating disorder) [9]. According to research on the relationship between IU and GAD [10], IU may develop and maintain symptoms of GAD by increasing repetitive negative thought (i.e., worry) [11]. Moreover, individuals with higher level of IU are more likely to treat ambiguous phenomena as unacceptable and threatening, which may lead to a negative problem orientation and an avoidance response style [12, 13]. Thus, they will be more prone to enter the process of worry. Under such model, increasing the patient’s tolerance and acceptance of uncertainty are the center of GAD therapy [14]. This strategy is supported by some randomized clinical trials with moderate to large effects [15–17].

To date, the pathways through how IU is related to individual symptoms of GAD still need to be further explored. Prior researches have generally studied IU at the disorder level or the core symptom level (i.e., worry) [18–23]. Studies have compared the IU across different diagnostic groups or examined IU in relation to total scores on self-report measures of GAD symptoms (such as Beck Anxiety Inventory [19], Trait-Anxiety Scale of State-Trait Anxiety Inventory [20], Hamilton Rating Scale for Anxiety [21], and Generalized Anxiety Disorder Questionnaire for the Diagnostic and Statistical Manual of Mental Disorders 4th edition [22]) or worry (such as Penn State Worry Questionnaire [18–21, 23]). However, as GAD is a heterogeneous syndrome characterized by different components of worry (e.g., excessiveness and uncontrollability components) and various cognitive, affective, and physical symptoms, the conclusions drew from these mentioned studies might be problematic. Moreover, IU is a complex construct, consisting of beliefs, emotions, and behaviors [11]. These different components of IU may play different roles in the development and maintenance of GAD. Thus, neglecting the different components of IU and symptomatic heterogeneity of GAD are serious limitations because it may mask differential associations between components of IU and different clinical symptoms. In order to further understanding the relationship between IU and GAD, a more fine-grained approach should be adopted considering different components of IU and symptoms of GAD.

A promising approach revealing complex relations among individual symptoms of mental disorders and their risk factor is the network approach. According to network approach, mental disorders arise from complex reciprocal influences between their constituting symptoms, instead of a latent common cause [24, 25]. Recently, research has expanded symptom networks [26–28]. The researchers integrate cognitive and biological factors that are considered as the causal roles in mental disorders, in order to find out the causality of risk factor and symptoms in mental disorders. A systematic review article has also demonstrated that adding non-symptom (e.g., risk factor) should enhance the understanding of important aspects of psychopathology [29]. In addition, this approach can give several centralities (e.g., strength and bridge strength) and predictability indicators for each node to quantify their importance and controllability in the entire network [29, 30].

By integrating similar self-report dispositional vulnerability factors (i.e., repetitive negative thinking and positive reappraisal) into symptom networks of depression and anxiety, research has found that these factors are differentially related to affective, cognitive, and somatic symptoms of depression and anxiety [31]. These differences cast light on potential pathways through which repetitive negative thinking and positive reappraisal may operate within depression and anxiety [31]. By incorporating genetic risk scores into symptom networks of psychosis, research has showed that the polygenic risk score is directly connected to the spectrum of positive and depressive symptoms and allowed for a novel outlook on the investigation of the relations between genome-wide association study-based polygenic risk scores and symptoms of mental disorders [32]. These studies supported that adding important and meaningful non-symptom components as nodes in related symptom networks is both empirically feasible and theoretically enriching [26, 29].

In the present study, we tend to put different components of IU and symptoms of GAD into one network. There were three aims in the present study. First, we want to investigate potential pathways between different components of IU and symptoms of GAD. Second, using centrality index to examine the relative importance of different components of IU and symptoms of GAD in the present network. Third, using bridge centrality index to examine which component of IU has the strongest connections with symptoms of GAD and which symptom of GAD has the strongest connections with different components of IU. In addressing these objectives, we sought to keep with the Research Domain Criteria [33] by considering varying degrees of different components of IU along the continuum of severity of different GAD symptoms. In this way, we attempted to improve the understanding of complex relations between IU and GAD.

## Methods

### Ethics statement

The Ethics Committee of the First Affiliated Hospital of the Fourth military medical university approved this study under the number No. KY20182047-F-1. With the help of Wenjuanxing (www.wjx.cn), we conducted this survey from 16 December 2020 to 18 December 2020. First the participants will read an informed consent. After they agreed to participate, they will finish the following items. Participants were also reminded that the survey was anonymous and personal information would not be disclosed.

### Participants

We used a dimensional approach that considered individual differences in different components of IU along a full range of normal to abnormal symptom severity levels of GAD (see Research Domain Criteria) [33]. Therefore, there were no eligibility requirements for participants in this study. Based on the fact of numerous active users of WeChat in China, it was used for the dissemination of this survey [34]. In the present network, we need to estimate 19 nodes and 171 edges. Although there are no clear guidelines yet as to how many participants we need per parameter, a rule of thumb put forward was at least three people per parameter [35]. Thus, the present network may need recruit at least 570 participants. A total of 633 university students from Xijing University participated in our study. All of these participants were Chinese speaking undergraduate students. Nine questionnaires were excluded due to their demographic information is incomplete. At last, a total of 624 questionnaires were obtained.

### Measures

#### Components of intolerance of uncertainty

The 12-item Intolerance of Uncertainty Scale (IUS-12) is a short, efficient, psychometrically sound scale for measuring IU [36, 37]. Items are rated on a five-point Likert scale ranging from 1 (“not at all characteristic of me”) to 5 (“entirely characteristic of me”). IUS-12 scores should be based on a simple sum of items, which the total score being used for evaluating a general IU [36]. In the present study, we used the Chinese version of IUS-12 to measure different components of IU [38]. The Chinese version of IUS-12 exhibits good reliability and validity. The Cronbach’s α is 0.88, and the 5-week reliability is 0.78. The Cronbach’s α of IUS-12 in this study was 0.84.

#### Symptoms of generalized anxiety disorder

The Generalized Anxiety Disorder 7-Item Questionnaire (GAD-7) is a valid and efficient self-report questionnaire for measuring the frequency of symptoms of GAD over the last 2 weeks [39]. GAD-7 has 7 items and each item varies from 0 to 3 (point referred to “not at all”, “several days”, “more than half the days”, and “nearly every day”, respectively). The sum of scores ranges from 0 to 21, and the higher the total score, the higher the level of GAD severity. In the present study, we used the Chinese version of GAD-7 to measure different symptoms of GAD [40]. The Chinese version of GAD-7 has good reliability and validity. The Cronbach’s α is in the range of 0.89 to 0.93 [40–42]. The Cronbach’s α of GAD-7 in this study was 0.90.

### Network analysis

We used Gaussian graphical model (GGM) to estimate the final network [43]. Within a GGM, the edges represent the partial correlation between nodes after controlling for all other nodes in the network. As recommended by Epskamp and Fried, we used the nonparametric Spearman rho correlations to estimate the network structure in order to account for the ordinal nature of the IUS-12 and GAD-7 [44]. The regularization of the GGM was conducted via the graphical lasso algorithm [45]. This regularization process shrunk all edges, and the edge with small partial correlation was shrunk to zero, thus the easier to interpret and more stable network can be obtained [44, 45]. At the same time, the tuning parameter was set to 0.5, which was a good balance between the sensitivity and specificity of extracting true edge [44, 46]. The visualization of the network was conducted by the Fruchterman-Reingold algorithm [47]. In the visualized network, the blue edge represents the positive correlation, while the red edge represents the negative correlation. A thicker edge means a stronger correlation between two connected nodes. The network was constructed and visualized using R-package *qgraph* [48].

The node expected influence was calculated via R-package *qgraph* [48]. Node expected influence is more appropriate for the network with both negative and positive edges when compared with the traditional node centrality index (e.g., node strength) [49]. Expected influence is defined as the sum of the value of all edges connecting to a given node. The higher the expected influence, the more important node is in the network. Moreover, the node bridge expected influence was computed by R-package *networktools* [50]. Bridge expected influence is defined as the sum of the value of all edges connecting a given node with nodes in the other community. Higher bridge expected influence values mean greater extent for increasing risk of contagion to other communities [50]. In the present network, we divided nodes into two communities in advance: one community is IU, which includes 12 components of IU (IUS-12) and the other community is GAD, which consists of seven anxiety symptoms (GAD-7). In addition, the predictability of node was calculated via R-package *mgm* [30]. Predictability represents the extent to which the variance of a node can be explained by all of its connected nodes.

The robustness of network was evaluated by R-package *bootnet* [51]. The accuracy of edge weight was evaluated by computing 95% confidence intervals using a non-parametric bootstrap approach with 2000 samples and calculating bootstrapped difference tests for edge weight. The stability of node expected influence and node bridge expected influence were evaluated via calculating correlation stability coefficient using a case-dropping bootstrap approach with 2000 samples, and by calculating bootstrapped difference tests for node expected influence and bridge expected influence. The value of correlation stability coefficient preferably should be above 0.5 and should not be below 0.25 [51].

In fact, recent bifactor analyses have found that in clinical and non-clinical samples the IUS-12 is best represented by a general factor, which explains most of the reliable variance across the items [52–55]. These findings indicate that a total score is an appropriate index of IU [9]. Thus, we also constructed one network consisting of eight nodes (i.e., one node represents the total score of IUS-12 and seven nodes represent the seven items of GAD-7) and calculated the related indexes.

## Results

### Descriptive statistics

The mean age of these 624 university students (43% male) was 19.38 ± 1.12 years (mean ± SD, range 18–25 years). In addition, 246 participants were sole offspring and 378 participants were non-sole offspring. Participants’ GAD-7 scores represented almost the full range of symptom severity. On the GAD-7 (M = 4.63, SD = 3.86), a total of 333 participants had minimal anxiety symptoms (range = 0–4), 236 had mild anxiety symptoms (range = 5–9), 37 had moderate anxiety symptoms (range = 10–14), and 18 had severe anxiety symptoms (range = 15–21). Furthermore, the scores on the IUS-12 (M = 35.08, SD = 7.44, range = 15–55) almost covered the full range of IU. Together, the distributional characteristics of the variables basically allowed the present investigation to estimate the strength of the relations among individual differences in different components of IU and symptoms of GAD. Table 1 shows abbreviation, mean scores, standard deviations and predictability for each variable selected in the present network. Table S1 showed the nonparametric Spearman rho correlation matrix of these variables (in Supplemental Material).

**Table 1:** Abbreviation, mean scores, standard deviations and predictability for each variable selected in the present network

Variables	Abbreviation	M	SD	Pre
Components of intolerance of uncertainty				
IUS-12-1: Unforeseen events upset me greatly	IU1	2.97	1.05	0.41
IUS-12-2: It frustrates me not having all the information I need	IU2	2.93	1.06	0.46
IUS-12-3: One should always look ahead so as to avoid surprises	IU3	3.52	0.94	0.13
IUS-12-4: A small, unforeseen event can spoil everything, even with the best of planning	IU4	2.90	1.01	0.26
IUS-12-5: I always want to know what the future has in store for me	IU5	3.23	1.09	0.22
IUS-12-6: I can’t stand being taken by surprise	IU6	2.81	1.01	0.40
IUS-12-7: I should be able to organize everything in advance	IU7	3.35	0.95	0.13
IUS-12-8: Uncertainty keeps me from living a full life	IU8	2.67	1.06	0.44
IUS-12-9: When it’s time to act, uncertainty paralyses me	IU9	2.80	1.08	0.44
IUS-12-10: When I am uncertain I can’t function very well	IU10	2.88	1.07	0.49
IUS-12-11: The smallest doubt can stop me from acting	IU11	2.58	1.07	0.52
IUS-12-12: I must get away from all uncertain situations	IU12	2.44	0.99	0.43
Symptoms of generalized anxiety disorder				
GAD-1: Feeling nervous, anxious or on edge	A1	0.89	0.65	0.48
GAD-2: Not being able to stop or control worrying	A2	0.66	0.66	0.52
GAD-3: Worrying too much about different things	A3	0.77	0.74	0.58
GAD-4: Trouble relaxing	A4	0.69	0.77	0.55
GAD-5: Being so restless that it is hard to sit still	A5	0.48	0.68	0.52
GAD-6: Becoming easily annoyed or irritable	A6	0.71	0.72	0.53
GAD-7: Feeling afraid as if something awful might happen	A7	0.43	0.64	0.52

*Abbreviations*: *M* mean, *SD* standard deviation, *Pre* predictability

### Network structure

**Fig. 1:**
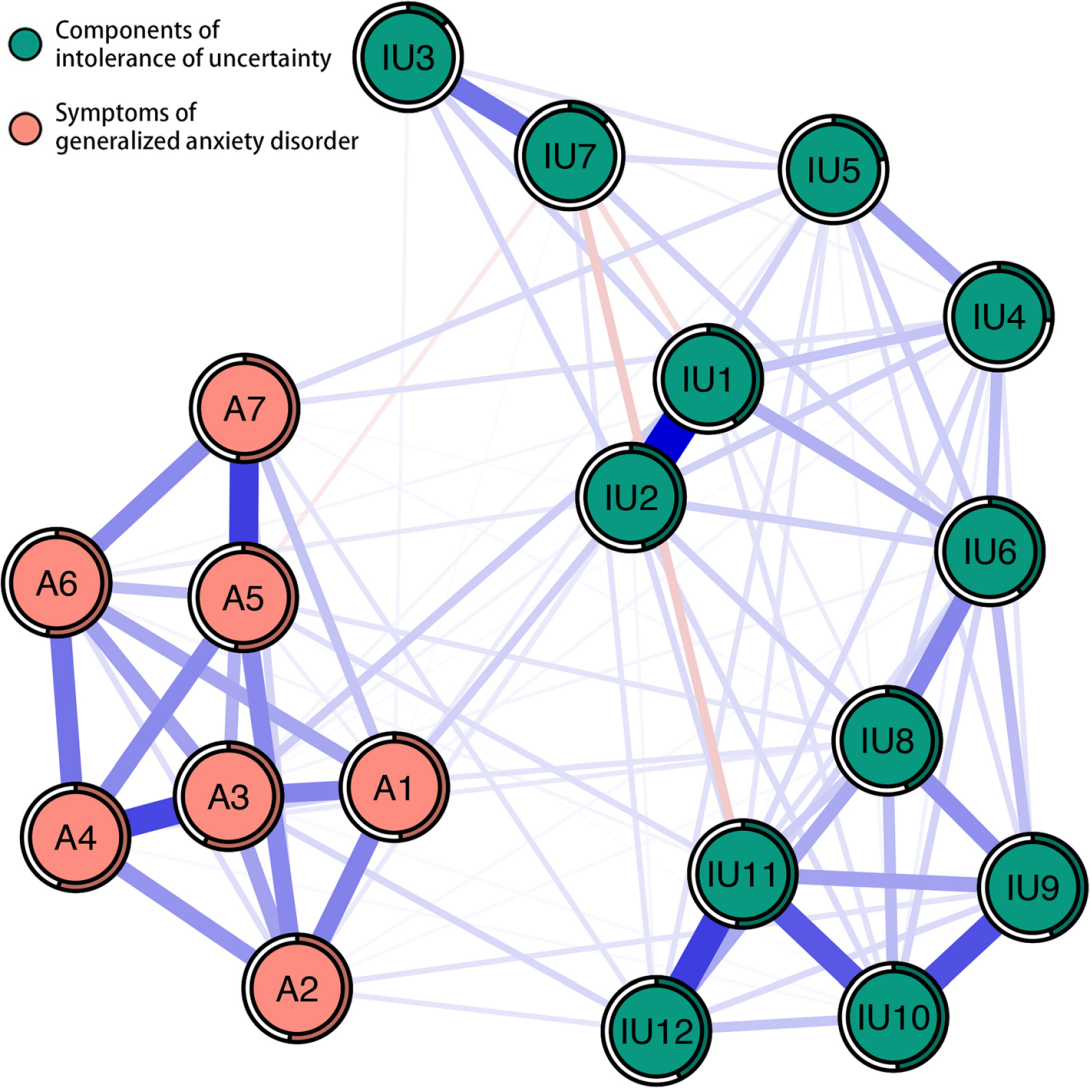
Network structure of different components of intolerance of uncertainty and symptoms of generalized anxiety disorder. Blue edges represent positive correlations, red edges represent negative correlations. The thickness of the edge reflects the magnitude of the correlation. The circles around nodes depict its predictability. IU1 = Unforeseen events upset me greatly; IU2 = It frustrates me not having all the information I need; IU3 = One should always look ahead so as to avoid surprises; IU4 = A small, unforeseen event can spoil everything, even with the best of planning; IU5 = I always want to know what the future has in store for me; IU6 = I can’t stand being taken by surprise; IU7 = I should be able to organize everything in advance; IU8 = Uncertainty keeps me from living a full life; IU9 = When it’s time to act, uncertainty paralyses me; IU10 = When I am uncertain I can’t function very well; IU11 = The smallest doubt can stop me from acting; IU12 = I must get away from all uncertain situations; A1 = Feeling nervous, anxious or on edge; A2 = Not being able to stop or control worrying; A3 = Worrying too much about different things; A4 = Trouble relaxing; A5 = Being so restless that it is hard to sit still; A6 = Becoming easily annoyed or irritable; A7 = Feeling afraid as if something awful might happen

Network structure of different components of IU and symptoms of GAD is shown in Fig. 1. This network shows some characteristics as below. First, 102 edges are not zero (60%) among 171 possible edges and most of these edges are positive except four negative edges. Second, we find six strongest edges in the final network. Four of the strongest edges are between IU’s components IU1 “Unforeseen events upset me greatly” and IU2 “It frustrates me not having all the information I need” (weight = 0.39), IU11 “The smallest doubt can stop me from acting” and IU12 “I must get away from all uncertain situations” (weight = 0.30), IU9 “When it’s time to act, uncertainty paralyses me” and IU10 “When I am uncertain I can’t function very well” (weight = 0.27), and IU10 “When I am uncertain I can’t function very well” and IU11 “The smallest doubt can stop me from acting” (weight = 0.26). Among anxiety symptoms, we find two strongest edges are between A5 “Being so restless that it is hard to sit still” and A7 “Feeling afraid as if something awful might happen” (weight = 0.30), and A3 “Worrying too much about different things” and A4 “Trouble relaxing” (weight = 0.28). It is noted that those strongest edges have no one which linked IU’s components and anxiety symptoms. Bootstrapped 95% confidence interval suggesting the accuracy of edge weights was relatively accurate and reliable (Figure S1 in the supplementary material). Moreover, in the present network, bootstrapped difference test for edge weights indicates that six strongest edge weights are significantly stronger than about 80 to 100% proportion of the other edge weights (Figure S2 in the supplementary material). Third, node predictability is depicted as circle around node in Fig. 1. The value of node predictability ranges from 13 to 58%, and the average is 42%. This indicates that on average, 42% of the variance of nodes in the present network can be explained by their neighboring nodes. Anxiety symptom A3 “Worrying too much about different things” has the highest predictability, indicating that 58% of its variance can be explained by its neighbors. And IU’s components IU7 “I should be able to organize everything in advance” and IU3 “One should always look ahead so as to avoid surprises” have the lowest predictability, indicating that both 13% of their variances can be explained by their neighbors (see Table 1).

**Fig. 2:**
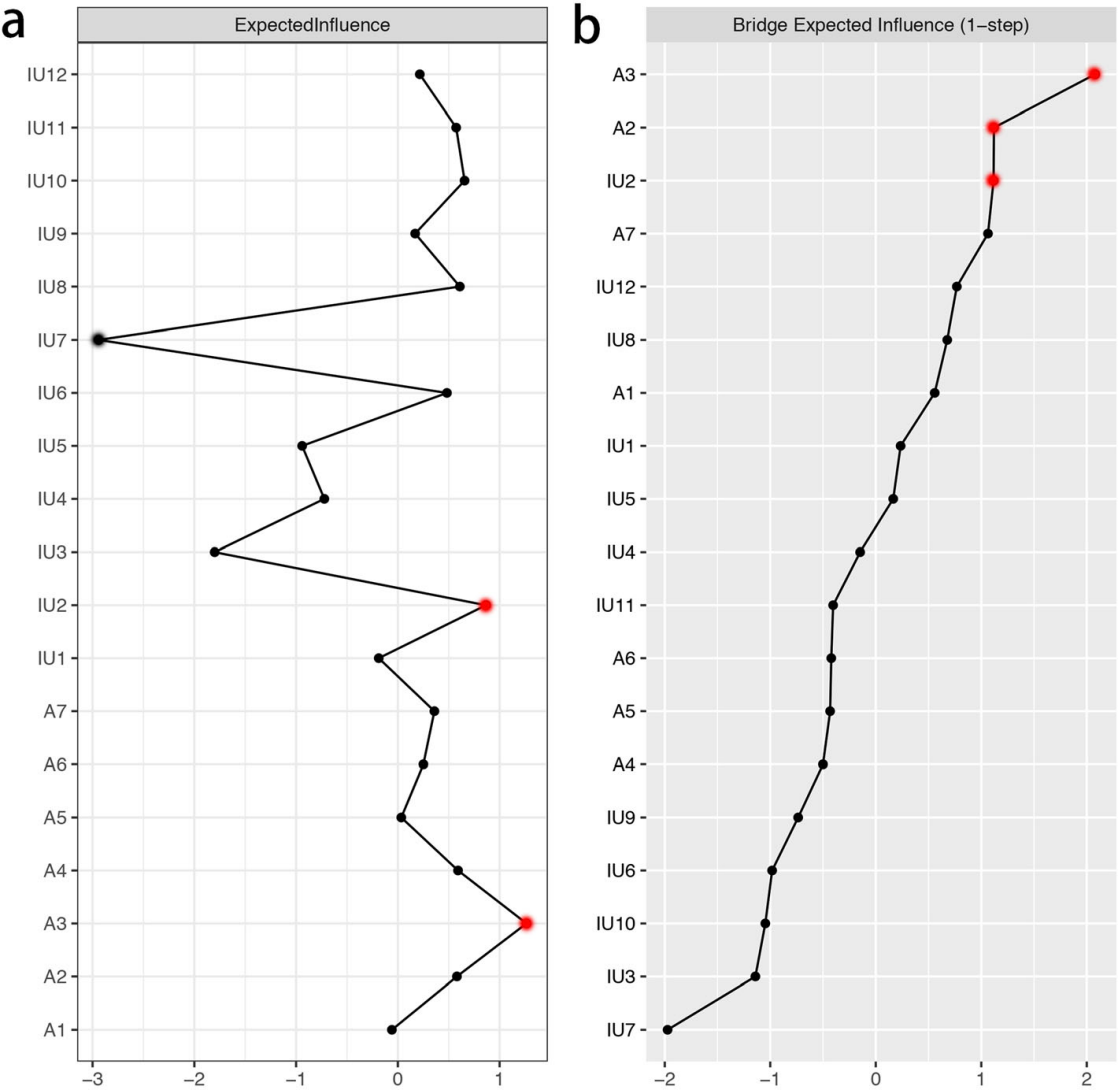
Centrality plot depicting the expected influence and bridge expected influence of each variable selected in the present network (z-score). IU1 = Unforeseen events upset me greatly; IU2 = It frustrates me not having all the information I need; IU3 = One should always look ahead so as to avoid surprises; IU4 = A small, unforeseen event can spoil everything, even with the best of planning; IU5 = I always want to know what the future has in store for me; IU6 = I can’t stand being taken by surprise; IU7 = I should be able to organize everything in advance; IU8 = Uncertainty keeps me from living a full life; IU9 = When it’s time to act, uncertainty paralyses me; IU10 = When I am uncertain I can’t function very well; IU11 = The smallest doubt can stop me from acting; IU12 = I must get away from all uncertain situations; A1 = Feeling nervous, anxious or on edge; A2 = Not being able to stop or control worrying; A3 = Worrying too much about different things; A4 = Trouble relaxing; A5 = Being so restless that it is hard to sit still; A6 = Becoming easily annoyed or irritable; A7 = Feeling afraid as if something awful might happen

The node expected influence is shown in Fig. 2a. A3 “Worrying too much about different things” and IU2 “It frustrates me not having all the information I need” have the highest expected influence, indicating that these two variables are the most associated nodes in the present network from the perspective of statistics. IU7 “I should be able to organize everything in advance” of IU has the lowest expected influence, indicating that this variable is the least associated node in the present network from the perspective of statistics. The correlation stability coefficient of expected influences is 0.75, indicating that the estimation of expected influences is adequately stable (Figure S3 in the supplementary material). Moreover, bootstrapped difference tests for expected influences show that in the present network, the expected influences of A3 “Worrying too much about different things” and IU2 “It frustrates me not having all the information I need” are significantly higher than about 30 to 60% proportion of the expected influences of other nodes and the expected influence of IU7 “I should be able to organize everything in advance” is significantly lower than all other nodes’ expected influences (Figure S4 in the supplementary material).

The node bridge expected influence is shown in Fig. 2b. In the community of IU, IU2 “It frustrates me not having all the information I need” has the highest bridge expected influence. This indicates that in the community of IU, IU2 “It frustrates me not having all the information I need” has the strongest connections with GAD symptoms. In the community of GAD, A3 “Worrying too much about different things” and A2 “Not being able to stop or control worrying” have the highest bridge expected influences. This indicates that in the community of GAD, these two symptoms have the strongest connections with IU’s components. The correlation stability coefficient of bridge expected influences is 0.44, indicating that the estimation of bridge expected influences meets the requirement (Figure S5 in the supplementary material). Bootstrapped difference tests for bridge expected influences show that in the present network, the bridge expected influences of A3 “Worrying too much about different things”, A2 “Not being able to stop or control worrying”, and IU2 “It frustrates me not having all the information I need” are significantly higher than about 30 to 60% proportion of the bridge expected influences of other nodes (Figure S6 in the supplementary material).

The network consisting of eight nodes (i.e., one node represents the total score of IUS-12 and seven nodes represent the seven items of GAD-7) shows that IU is directly related to all GAD symptoms. The relations between IU and A3 “Worrying too much about different things” and A2 “Not being able to stop or control worrying” are the strongest relations. Readers interested in this network can find related results in the supplementary materials (Table S2 and Figure S7).

## Discussion

Employing network analysis, we aim to reveal potential pathways that how different components of IU are related to symptoms of GAD. It is observed that different components of IU are commonly but differentially related to symptoms of GAD. These results suggest that different components of IU may have similar and specific pathways to develop and maintain GAD. In some extent, this finding adds to emerging research showing that cognitive risk factors differ considerably for individual symptoms [27, 31, 56, 57].

The strongest edges exist within each community, which is aligned with the previous network studies consisting of two communities (i.e., one community consists of depression symptoms and the other community consists of anxiety symptoms) [31, 58–62]. Within components of IU, the present study finds a strongest edge exists between IU1 “Unforeseen events upset me greatly” and IU2 “It frustrates me not having all the information I need”, which is similar to the results of previous networks of IU [63]. In fact, these two components are emotional reactions to uncertainty. This edge also supports the notion that not knowing is highly related to aversive emotional outcomes (i.e., upset and frustration) [63]. The other three strongest edges are between IU11 “The smallest doubt can stop me from acting” and IU12 “I must get away from all uncertain situations”, between IU9 “When it’s time to act, uncertainty paralyses me” and IU10 “When I am uncertain I can’t function very well”, and between IU10 “When I am uncertain I can’t function very well” and IU11 “The smallest doubt can stop me from acting”. These four components reflect the sense of feeling “stuck” and unable to respond when faced with uncertainty and the fact that uncertainty causes functional impairments, so that it has to be avoided. Results about these three edges are similar with the results of a previous research applying network analysis to investigate the internal structure of IU [63]. Within symptoms of GAD, the present study finds two strongest edges. One edge is between A5 “Being so restless that it is hard to sit still” and A7 “Feeling afraid as if something awful might happen”, which is consistent with the results of previous research investigating network structure of depression and anxiety symptoms in Chinese female nursing students [59]. The other edge is between A3 “Worrying too much about different things” and A4 “Trouble relaxing”, which is aligned with the results of previous network researches investigating comorbidity of depression and anxiety symptoms in migrant Filipino domestic workers [60]. It should be noted that the relations between components of IU and symptoms of GAD are relatively small. Thus, the contribution of IU to GAD should not be overstated.

Symptom A3 “Worrying too much about different things” of GAD and component IU2 “It frustrates me not having all the information I need” of IU have the highest expected influences which indicates these two variables may play the most important role in activating and maintaining the present network consisting of 12 components of IU and seven symptoms of GAD. A previous study found that IU7 “I should be able to organize everything in advance” is the central components of IU in both undergraduate and community population [63]. However, in the current study, component IU7 “I should be able to organize everything in advance” has the lowest centrality. And all of the only four negative edges in the network include IU7 “I should be able to organize everything in advance”. This difference may be caused by cultural differences. In Chinese culture, organizing things in advance is a mature performance, which may represent the positive response rather than intolerance when facing uncertainty. This supports the viewpoint that cross-cultural differences may occur in the interpretation of components of IU [52].

In the current network, node bridge strength centrality may cast light on the specific role played by these different components of IU in the context of GAD. In the community of IU, component IU2 “It frustrates me not having all the information I need” has the highest bridge expected influence. This suggests that IU2 “It frustrates me not having all the information I need” has stronger associations with symptoms of GAD than other components. Thus, from a network perspective, targeting component IU2 “It frustrates me not having all the information I need” may be more effective at reducing symptoms of GAD than targeting other components of IU. It is worth mentioning that this represents a hypothesis, which should be tested in an experimental and clinical manner [64]. From a conceptual perspective, the uncertain situation may lead individuals to seek as much information as possible in order to cope with uncertainty. However, it is extremely difficult to know all the information. Under this circumstance, individuals may begin to have negative evaluations of uncertain situation and yield negative emotions and maladaptive coping behaviors, which in return foster symptoms of anxiety. In the community of GAD, symptoms A3 “Worrying too much about different things” and A2 “Not being able to stop or control worrying” have the higher bridge expected influences than other symptoms. This suggests that these two symptoms have stronger associations with components of IU. In fact, these two symptoms belong to critical aspects of worry (i.e., excessiveness and uncontrollability) [65]. Previous studies have found that there is a strong correlation between IU and worry [66, 67]. IU is important in both creating a worry bout and maintaining it [68] and, the strong relationship between IU and worry has been replicated in numerous studies [18–20, 66, 69]. Moreover, a longitudinal study in adolescents found a bidirectional and reciprocal relationship between IU and worry in which change in one variable partially explained change in the other [70]. Previous study using ecological momentary assessment also found that IU was significantly associated with worry [13]. Our results verify the relations between worry and IU from a network perspective.

The present study provides a fine-grained understanding of the relations between different components of IU and symptoms of GAD. Symptom “Worrying too much about different things” and component “It frustrates me not having all the information I need” have the highest expected influences in the present network. In the community of IU, component “It frustrates me not having all the information I need” has the highest bridge expected influence. And in the community of GAD, symptoms “Worrying too much about different things” and “Not being able to stop or control worrying” have the highest bridge expected influence. These results may provide several implications for related preventions and interventions to meet the needs of mental health in Chinese university students, such as targeting IU2 “It frustrates me not having all the information I need” may be more effective at reducing symptoms of GAD than targeting other components of IU.

There are several limitations in the present study. First, we recruited Chinese university students and reporting components of IU and symptoms of GAD that span the full range of normal to abnormal, which likely limits the universality of our conclusions. The resulting network structure and related indicators (such as node expected influence and bridge expected influence) could differ when examined in clinical sample. Second, using cross-sectional data to obtain the network structure of components of IU and symptoms of GAD preclude claims about causality. Future studies could use longitudinal data to examine the causality of these variables. Third, the present network investigated between-subject effects on a group level. That is, the network structure of a single individual may not be replicated in the same way. Fourth, in the present study, the symptoms were single-item, self-reported assessments, which may be limited to capture clinical phenomena. Self-report data might be susceptible to shared method variance and subjective response biases, which can inflate relations between variables. Future studies could use more items and methods. Finally, the network structure in the present study is specific to the questionnaires we used. In fact, as suggested by a reviewer, GAD-7 only partially matches the diagnostic criteria of GAD and demonstrates poor specificity for all anxiety disorders [2, 39, 71]. Thus, the interpretation of the results should be cautious.

## Conclusion

In conclusion, this study elucidates potential pathways between different components of IU and symptoms of GAD. Indeed, these novel findings highlight how including hypothesized risk factors may enrich symptom networks to gain a fine-grained understanding of processes operating in mental disorders. Understanding how putative risk factors such as different components of IU are related to symptoms of GAD may provide some references for related preventions and interventions, such as targeting component “It frustrates me not having all the information I need” may be more effective at reducing symptoms of GAD than targeting other components of IU.

## Supplementary Information

**Additional file 1.**

## Data Availability

The datasets used and/or analyzed during the current study are available from the corresponding author on reasonable request.

## Abbreviations

IU: Intolerance of uncertainty
GAD: Generalized Anxiety Disorder
IUS-12: 12-item Intolerance of Uncertainty Scale
GAD-7: Generalized Anxiety Disorder 7-Item Questionnaire
GGM: Gaussian graphical model
IU1: Unforeseen events upset me greatly
IU2: It frustrates me not having all the information I need
IU3: One should always look ahead so as to avoid surprises
IU4: A small, unforeseen event can spoil everything, even with the best of planning
IU5: I always want to know what the future has in store for me
IU6: I can’t stand being taken by surprise
IU7: I should be able to organize everything in advance
IU8: Uncertainty keeps me from living a full life
IU9: When it’s time to act, uncertainty paralyses me
IU10: When I am uncertain I can’t function very well
IU11: The smallest doubt can stop me from acting
IU12: I must get away from all uncertain situations
A1: Feeling nervous, anxious or on edge
A2: Not being able to stop or control worrying
A3: Worrying too much about different things
A4: Trouble relaxing
A5: Being so restless that it is hard to sit still
A6: Becoming easily annoyed or irritable
A7: Feeling afraid as if something awful might happen
M: Mean
SD: Standard deviation
Pre: Predictability
