# Supplementary material for: The relations between different components of intolerance of uncertainty and symptoms of generalized anxiety disorder: a network analysis
Source: BMC Psychiatry. 2021 Sep 10;21:448. doi: 10.1186/s12888-021-03455-0 (PMC8431915; doi:10.1186/s12888-021-03455-0)
Supplement: Supplementary file 1 — Additional file 1. [file 12888_2021_3455_MOESM1_ESM.docx]

**The relations between different components of intolerance of uncertainty and symptoms of generalized anxiety disorder: a network analysis**

Lei Ren^1†^, Zihan Wei^2†^, Ye Li^3^, Long-Biao Cui^1^, Yifei Wang^1^, Lin Wu^1^, Xinyi Wei^4^, Jiaxi Peng^5^, Kuiliang Li^6^, Yinchuan Jin^1^, Fengzhan Li^1^, Qun Yang^1*^, Xufeng Liu^1*^

^1^Department of Military Medical Psychology, Air Force Medical University, 710032, Xi’an, China

^2^Department of Neurology, Xijing Hospital, Air Force Medical University, 710032, Xi’an, China

^3^Psychological counseling center, Xijing University, 710100, Xi’an, China

^4^Department of Psychology, Renmin University of China, 100000, Beijing, China

^5^College of Teachers, Chengdu University, 610106, Chengdu, China

^6^Department of Psychology, Army Medical University, 400038, Chongqing, China

^*^ Correspondence: yangqun1125@hotmail.com; lxf_fmmu@163.com

^†^ Lei Ren and Zihan Wei contributed equally to this work

**Supplementary Materials**

1. Table S1. Nonparametric Spearman rho correlation matrix of variables selected in the present network
2. Figure S1. Accuracy of edge weights
3. Figure S2. Bootstrapped difference test for edge weights
4. Figure S3. Stability of node expected influences
5. Figure S4. Bootstrapped difference test for node expected influences
6. Figure S5. Stability of node bridge expected influences
7. Figure S6. Bootstrapped difference test for node bridge expected influences
8. Table S2. Regularized partial correlation matrix of variables selected in the network consisting of eight nodes
9. Figure S7. The related results of the network consisting of eight nodes

Table S1. Nonparametric Spearman rho correlation matrix of variables selected in the present network

|  | IU1 | IU2 | IU3 | IU4 | IU5 | IU6 | IU7 | IU8 | IU9 | IU10 | IU11 | IU12 | A1 | A2 | A3 | A4 | A5 | A6 | A7 |
| --- | --- | --- | --- | --- | --- | --- | --- | --- | --- | --- | --- | --- | --- | --- | --- | --- | --- | --- | --- |
| IU1 | 1.00 |  |  |  |  |  |  |  |  |  |  |  |  |  |  |  |  |  |  |
| IU2 | 0.58^**^ | 1.00 |  |  |  |  |  |  |  |  |  |  |  |  |  |  |  |  |  |
| IU3 | 0.19^**^ | 0.20^**^ | 1.00 |  |  |  |  |  |  |  |  |  |  |  |  |  |  |  |  |
| IU4 | 0.34^**^ | 0.35^**^ | 0.13^**^ | 1.00 |  |  |  |  |  |  |  |  |  |  |  |  |  |  |  |
| IU5 | 0.21^**^ | 0.29^**^ | 0.15^**^ | 0.31^**^ | 1.00 |  |  |  |  |  |  |  |  |  |  |  |  |  |  |
| IU6 | 0.40^**^ | 0.40^**^ | 0.12^**^ | 0.36^**^ | 0.30^**^ | 1.00 |  |  |  |  |  |  |  |  |  |  |  |  |  |
| IU7 | -0.05 | -0.02 | 0.25^**^ | 0.04 | 0.11^**^ | 0.10^*^ | 1.00 |  |  |  |  |  |  |  |  |  |  |  |  |
| IU8 | 0.36^**^ | 0.40^**^ | 0.10^*^ | 0.34^**^ | 0.26^**^ | 0.49^**^ | 0.02 | 1.00 |  |  |  |  |  |  |  |  |  |  |  |
| IU9 | 0.25^**^ | 0.31^**^ | 0.09^*^ | 0.31^**^ | 0.28^**^ | 0.43^**^ | 0.01 | 0.50^**^ | 1.00 |  |  |  |  |  |  |  |  |  |  |
| IU10 | 0.35^**^ | 0.39^**^ | 0.15^**^ | 0.31^**^ | 0.27^**^ | 0.43^**^ | 0.01 | 0.49^**^ | 0.57^**^ | 1.00 |  |  |  |  |  |  |  |  |  |
| IU11 | 0.31^**^ | 0.38^**^ | 0.06 | 0.33^**^ | 0.29^**^ | 0.42^**^ | -0.08^*^ | 0.49^**^ | 0.52^**^ | 0.58^**^ | 1.00 |  |  |  |  |  |  |  |  |
| IU12 | 0.27^**^ | 0.34^**^ | 0.12^**^ | 0.29^**^ | 0.28^**^ | 0.40^**^ | 0.06 | 0.47^**^ | 0.44^**^ | 0.47^**^ | 0.57^**^ | 1.00 |  |  |  |  |  |  |  |
| A1 | 0.27^**^ | 0.34^**^ | 0.12^**^ | 0.24^**^ | 0.23^**^ | 0.25^**^ | -0.04 | 0.29^**^ | 0.25^**^ | 0.26^**^ | 0.25^**^ | 0.24^**^ | 1.00 |  |  |  |  |  |  |
| A2 | 0.31^**^ | 0.34^**^ | 0.09^*^ | 0.24^**^ | 0.20^**^ | 0.29^**^ | -0.04 | 0.33^**^ | 0.30^**^ | 0.28^**^ | 0.30^**^ | 0.33^**^ | 0.54^**^ | 1.00 |  |  |  |  |  |
| A3 | 0.35^**^ | 0.36^**^ | 0.09^*^ | 0.27^**^ | 0.24^**^ | 0.30^**^ | 0.02 | 0.36^**^ | 0.29^**^ | 0.31^**^ | 0.31^**^ | 0.35^**^ | 0.56^**^ | 0.57^**^ | 1.00 |  |  |  |  |
| A4 | 0.28^**^ | 0.31^**^ | 0.08^*^ | 0.23^**^ | 0.17^**^ | 0.28^**^ | 0.03 | 0.34^**^ | 0.26^**^ | 0.28^**^ | 0.29^**^ | 0.30^**^ | 0.50^**^ | 0.57^**^ | 0.64^**^ | 1.00 |  |  |  |
| A5 | 0.19^**^ | 0.28^**^ | 0.06 | 0.19^**^ | 0.20^**^ | 0.22^**^ | -0.07 | 0.32^**^ | 0.25^**^ | 0.28^**^ | 0.32^**^ | 0.30^**^ | 0.44^**^ | 0.54^**^ | 0.46^**^ | 0.55^**^ | 1.00 |  |  |
| A6 | 0.27^**^ | 0.32^**^ | 0.07 | 0.24^**^ | 0.16^**^ | 0.24^**^ | -0.04 | 0.27^**^ | 0.24^**^ | 0.23^**^ | 0.29^**^ | 0.25^**^ | 0.51^**^ | 0.50^**^ | 0.57^**^ | 0.59^**^ | 0.51^**^ | 1.00 |  |
| A7 | 0.21^**^ | 0.30^**^ | 0.06 | 0.26^**^ | 0.25^**^ | 0.24^**^ | 0.02 | 0.31^**^ | 0.22^**^ | 0.25^**^ | 0.27^**^ | 0.31^**^ | 0.47^**^ | 0.49^**^ | 0.52^**^ | 0.48^**^ | 0.58^**^ | 0.54^**^ | 1.00 |

^**^ *p* < 0.01 (two-tailed); ^*^ *p* < 0.01 (two-tailed).

Figure S1. Accuracy of edge weights

*Note*: The red line depicts the sample edge weights and the gray bar depicts the bootstrapped confidence interval.

Figure S2. Bootstrapped difference test for edge weights

*Note*: Gray boxes indicate edge weights that do not differ significantly from one another, while black boxes indicate edge weights that do differ significantly. Blue and red boxes on the diagonal correspond to edge weights with positive and negative correlations, respectively.

Figure S3. Stability of node expected influences

*Note*: The red bar represents the average correlation between node expected influences in the full sample and subsample with the red area depicting the 2.5th quantile to the 97.5th quantile.

Figure S4. Bootstrapped difference test for node expected influences

*Note*: Gray boxes indicate node expected influences that do not differ significantly from one another, while black boxes indicate node expected influences that do differ significantly. The number in the white boxes (i.e., diagonal line) represent the value of node expected influences.

Figure S5. Stability of node bridge expected influences

*Note*: The red bar represents the average correlation between node bridge expected influences in the full sample and subsample with the red area depicting the 2.5th quantile to the 97.5th quantile.

Figure S6. Bootstrapped difference test for node bridge expected influences

*Note*: Gray boxes indicate node bridge expected influences that do not differ significantly from one another, while black boxes indicate node bridge expected influences that do differ significantly.

Table S2. Regularized partial correlation matrix of variables selected in the network consisting of eight nodes

|  | IUT | A1 | A2 | A3 | A4 | A5 | A6 | A7 |
| --- | --- | --- | --- | --- | --- | --- | --- | --- |
| IUT | 1.00 |  |  |  |  |  |  |  |
| A1 | 0.07 | 1.00 |  |  |  |  |  |  |
| A2 | 0.12 | 0.20 | 1.00 |  |  |  |  |  |
| A3 | 0.17 | 0.19 | 0.15 | 1.00 |  |  |  |  |
| A4 | 0.06 | 0.06 | 0.16 | 0.29 | 1.00 |  |  |  |
| A5 | 0.05 | 0.02 | 0.19 | 0.00 | 0.18 | 1.00 |  |  |
| A6 | 0.01 | 0.15 | 0.05 | 0.15 | 0.22 | 0.11 | 1.00 |  |
| A7 | 0.08 | 0.10 | 0.07 | 0.12 | 0.00 | 0.31 | 0.19 | 1.00 |


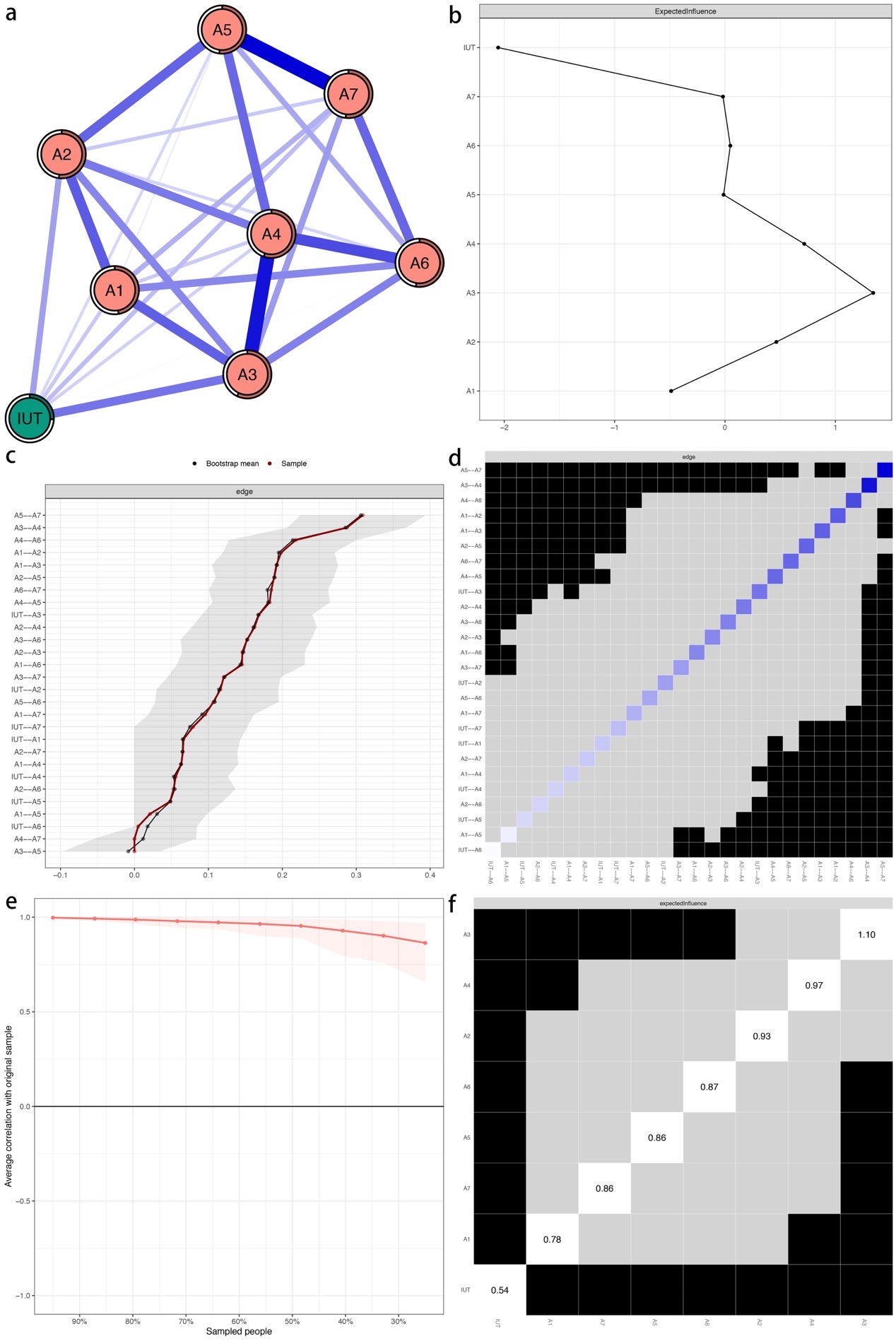


Figure S7. The related results of the network consisting of eight nodes

*Note*: IUT = Total score of IUS-12; (e) The correlation stability coefficient of expected influence is 0.75.
